# Reproductive Toxicity: Birth Weight Raises More Questions on Seafood Safety

**DOI:** 10.1289/ehp.116-a20

**Published:** 2008-01

**Authors:** Adrian Burton

Women who eat too much shellfish before pregnancy, particularly crabs and lobsters, may increase their chance of having babies who are small for their gestational age (SGA), report French scientists in an article posted online 24 October 2007 ahead of print in *Environmental Health*. Eating fish, however, seems to have the opposite effect. The findings further fuel the debate over how much and what types of fish and other seafood are beneficial to would-be moms.

“Some studies suggest the omega-3 fatty acids in fish and seafood are beneficial to fetal growth and birth weight,” explains first author Laurence Guldner, an epidemiologist at the National Institute of Health and Medical Research, University of Rennes, “but others report no benefit or even a negative effect.” The new report could help explain these discrepancies, because the results distinguish between the effects of fish and shellfish, and between even more specific subcategories of seafood—something most earlier work did not do.

The study included 2,398 pregnant women in Brittany, France, who were part of the Pélagie cohort assembled to investigate effects of environmental pollutants on pregnancy, birth outcomes, and child health and development. The researchers gathered information on consumption in the year prior to pregnancy of saltwater fish (e.g., salmon), mollusks (e.g., oysters), large crustaceans (e.g., lobster), and small crustaceans (e.g., shrimp).

Statistical analysis, adjusted for a number of potential confounders, showed that women who ate 2 or more meals of shellfish per week had a statistically significant 2.14 greater likelihood of having an SGA baby (defined as having birth weight below the tenth percentile for a given gestational age and sex) compared with those who ate shellfish less than once per month. Those who ate fish 2 or more times per week were about half as likely to have an SGA baby than those who ate it less than once per month (a nonsignificant finding).

“Most of the negative effect of seafood on SGA was [associated with] eating large crustaceans, like crabs and lobsters,” explains Guldner. He suggests that high tissue concentrations of persistent organic pollutants such as dioxins and polychlorinated biphenyls accumulated by these animals may cancel out the potential beneficial effect of their omega-3 fatty acids.

Indeed, the results of some other studies have suggested that low-level exposure *in utero* to such pollutants may have a negative effect on birth weight. However, the evidence to date is inconclusive.

“Unfortunately, no distinction was made between the fish types eaten [in this study],” cautions Thorhallur Ingi Halldorsson, a researcher at the State Serum Institute in Copenhagen, Denmark. “We should [therefore] be careful in promoting fish as beneficial for fetal growth. Oily fish is a good source of omega-3 fatty acids, but high consumption can lead to higher body burdens of organic pollutants, which might affect growth.” Halldorsson adds that regular consumption of varying fish types should therefore be encouraged.

Rosa Ortega, a professor of nutrition at the Complutense University of Madrid, Spain, adds, “It would certainly be a good idea for women to make sure their seafood comes from guaranteed clean waters. But with everything in moderation, a new mother-to-be is probably still safe to satisfy her craving for a lobster dinner.”

## Figures and Tables

**Figure:**
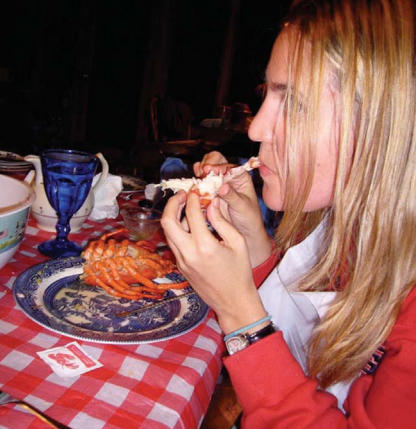
Eat now, pay later? Although it’s too soon to put the kibosh on lobster, a seafood study suggests that high crustacean consumption may be linked to low birth weight.

